# 1,3-Bis(3-*tert*-butyl-2-hy­droxy-5-meth­oxy­benz­yl)hexa­hydro­pyrimidin-5-ol monohydrate

**DOI:** 10.1107/S1600536814010769

**Published:** 2014-05-21

**Authors:** Augusto Rivera, Ingrid Miranda-Carvajal, Héctor Jairo Osorio, Jaime Ríos-Motta, Michael Bolte

**Affiliations:** a Universidad Nacional de Colombia, Sede Bogotá, Facultad de Ciencias, Departamento de Química, Cra 30 No. 45-03, Bogotá, Código Postal 111321, Colombia; b Universidad Nacional de Colombia, Sede Manizales, Colombia; c Institut für Anorganische Chemie, J. W. Goethe-Universität Frankfurt, Max-von-Laue-Str. 7, 60438 Frankfurt/Main, Germany

## Abstract

The asymmetric unit of the title compound, C_28_H_42_N_2_O_5_·H_2_O, consists of one half of the organic mol­ecule and one half-mol­ecule of water, both of which are located on a mirror plane which passes through the central C atoms and the hydroxyl group of the heterocyclic system. The hydroxyl group at the central ring is disordered over two equally occupied positions. The six-membered ring adopts a chair conformation, and the 2-hy­droxy­benzyl substituents occupy the sterically preferred equatorial positions. The aromatic rings make dihedral angles of 75.57 (9)° with the mean plane of the heterocyclic ring. The dihedral angle between the two aromatic rings is 19.18 (10)°. The mol­ecular structure features two intra­molecular phenolic O—H⋯N hydrogen bonds with graph-set motif *S*(6). In the crystal, mol­ecules are connected *via* O—H⋯O hydrogen bonds into zigzag chains running along the *a-*axis direction.

## Related literature   

For related structures, see: Rivera *et al.* (2012 ▶), Zhang *et al.* (2012 ▶). For the synthesis, see: Rivera *et al.* (2013 ▶). For bond-length data, see: Allen *et al.* (1987 ▶). For puckering parameters, see: Cremer & Pople (1975 ▶). For hydrogen-bond graph-set nomenclature, see: Bernstein *et al.* (1995 ▶).
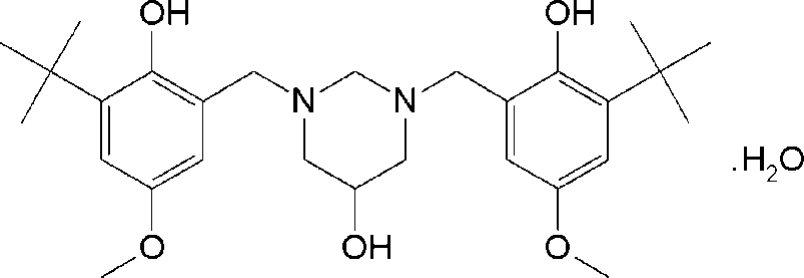


## Experimental   

### 

#### Crystal data  

C_28_H_42_N_2_O_5_·H_2_O
*M* *_r_* = 504.65
Orthorhombic, 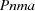

*a* = 8.2629 (5) Å
*b* = 33.093 (3) Å
*c* = 10.0877 (6) Å
*V* = 2758.4 (3) Å^3^
*Z* = 4
Mo *K*α radiation
μ = 0.09 mm^−1^
*T* = 173 K
0.25 × 0.23 × 0.16 mm

#### Data collection  

STOE IPDS II two-circle-diffractometer
Absorption correction: multi-scan (*X-AREA*; Stoe & Cie, 2001 ▶) *T*_min_ = 0.975, *T*_max_ = 0.982
22328 measured reflections
2464 independent reflections
2228 reflections with *I* > 2σ(*I*)
*R*_int_ = 0.109

#### Refinement  

*R*[*F* ^2^ > 2σ(*F* ^2^)] = 0.075
*wR*(*F* ^2^) = 0.140
*S* = 1.17
2464 reflections
179 parameters
H atoms treated by a mixture of independent and constrained refinement
Δρ_max_ = 0.26 e Å^−3^
Δρ_min_ = −0.31 e Å^−3^

### 

Data collection: *X-AREA* (Stoe & Cie, 2001 ▶); cell refinement: *X-AREA*; data reduction: *X-AREA* (Stoe & Cie, 2001 ▶); program(s) used to solve structure: *SHELXS2013* (Sheldrick, 2008 ▶); program(s) used to refine structure: *SHELXL2013* (Sheldrick, 2008 ▶); molecular graphics: *XP* in *SHELXTL* (Sheldrick, 2008 ▶) and *Mercury* (Macrae *et al.*, 2008 ▶); software used to prepare material for publication: *SHELXL2013*.

## Supplementary Material

Crystal structure: contains datablock(s) I, New_Global_Publ_Block. DOI: 10.1107/S1600536814010769/sj5401sup1.cif

Structure factors: contains datablock(s) I. DOI: 10.1107/S1600536814010769/sj5401Isup2.hkl

Supporting information file. DOI: 10.1107/S1600536814010769/sj5401Isup3.cml

CCDC reference: 1002201

Additional supporting information:  crystallographic information; 3D view; checkCIF report

## Figures and Tables

**Table 1:** Hydrogen-bond geometry (Å, °)

*D*—H⋯*A*	*D*—H	H⋯*A*	*D*⋯*A*	*D*—H⋯*A*
O1—H1⋯N1	0.94 (3)	1.80 (3)	2.671 (2)	153 (3)
O3—H3⋯O1*W*	0.89 (6)	1.93 (6)	2.813 (4)	174 (5)
O1*W*—H1*W*⋯O1^i^	0.84	2.19	3.029 (2)	173

Symmetry code: (i) 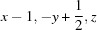.

**Fig. 1.**
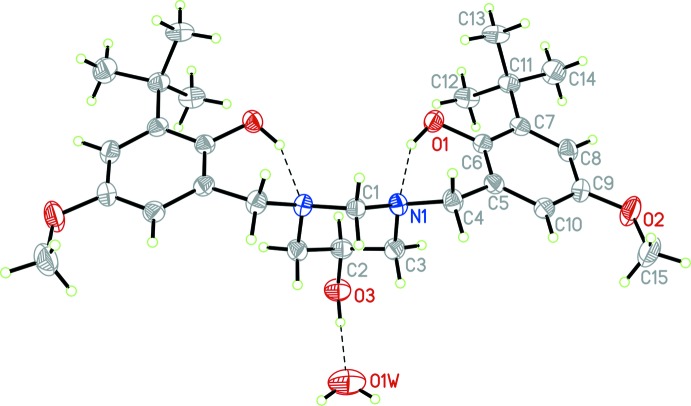
The molecular structure of the title compound, Displacement ellipsoids are drawn at the 50% probability level. Hydrogen bonds are drawn as dashed lines. Only one of the two sites of the disordered hydroxyl group is shown. Symmetry operator for generating equivalent atoms. x, 1/2 - y, z.

**Fig. 2.**
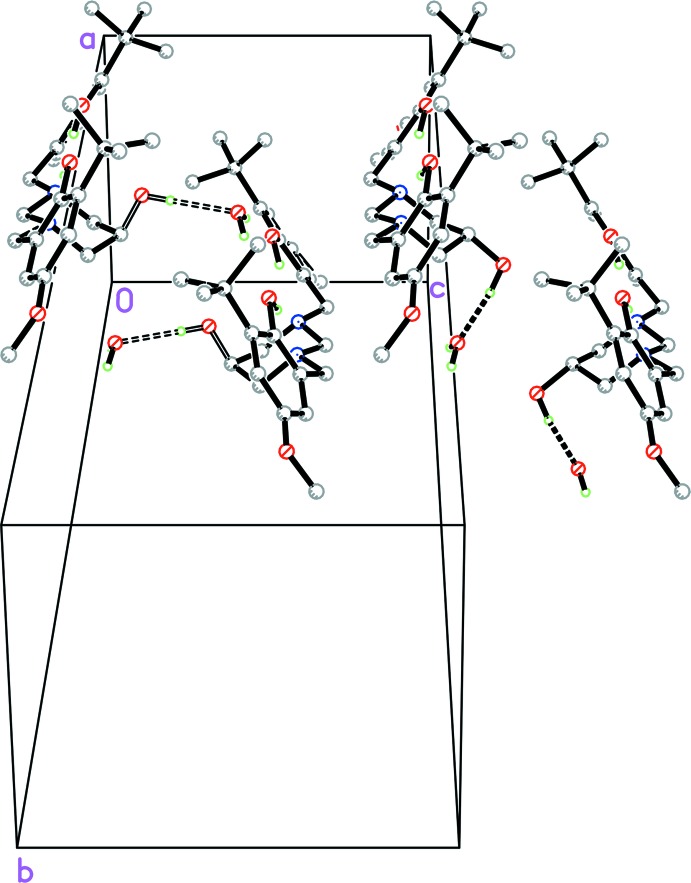
Partial packing diagram of the title compound. The bonds of the second hydroxyl group site (on the left) are drawn with open bonds. Hydrogen bonds involving the two different sites of the disordered hydroxy groups are drawn with full (right hand side) or open dashed (left hand side) bonds.

## supplementary crystallographic information

### 1. Comment

Recently our group reported a protocol for the synthesis of a series of 2,2'-(dihydropyrimidine-1,3(2*H*,4*H*)-diyldimethanediyl)diphenols *via* a Mannich-type reaction (Rivera *et al.*, 2013). As an extension of these studies, in this paper we describe the synthesis and crystal structure of the title compound, 1,3-bis(3-*tert*-butyl-2-hydroxy-5-methoxybenzyl)hexahydropyrimidin-5-ol, C_28_H_42_N_2_O_5_, as a monohydrate. The molecular structure and atom-numbering scheme for the title compound are shown in Fig. 1. The asymmetric unit contains one half-organic molecule and one half water molecule, which are both located on a mirror plane which passes through the central C atoms and the hydroxyl group of the heterocyclic system. In Fig. 1, atoms without labels were generated by the symmetry operator *x*, 1/2 - *y*, *z*. The 1,3-diazinane ring of the title compound adopts a chair conformation with a diequatorial substitution with puckering parameters Q, θ and φ of 0.594 (2) Å, 2.23 (19)°, 60 (4)° (Cremer & Pople, 1975). The aromatic rings make dihedral angles of 75.57 (9)° with the mean plane of the heterocyclic ring. The benzyl groups are located in a 1,3-diequatorial *syn* arrangement in the heterocyclic ring with a dihedral angle between the planes containing the aromatic rings of 19.18 (10)°. In the molecule of the title compound (Fig. 1), bond lengths (Allen *et al.*, 1987) and angles are normal and comparable to those observed in related structures namely 4,4',6,6'-tetra-*tert*-butyl-2,2'- [1,3-diazinane-1,3-diylbis(methylene)]diphenol 0.25-hydrate (Zhang *et al.*, 2012) and 6,6'-di-*tert*-butyl-4,4'-dimethoxy-2,2'- [1,3-diazinane-1,3-diylbis(methylene)]- diphenol 0.19-hydrate (Rivera *et al.*, 2012). The crystal structure shows two intramolecular O—H···N(1,3-diazinane) hydrogen bonds with graph-set motif S(6) (Bernstein *et al.*, 1995) (Table 1), where the N···O distance [N1···O1, 2.671 (2) Å] is shorter in comparison with the values observed in the related structure (Zhang *et al.* 2012). In contrast to the 4',6'-di-*tert*-butyl analog, the title compound was found to be more similar to the other related structure (Rivera *et al.*, 2012), indicating that the methoxy substituent slightly influences the strength of the intermolecular hydrogen bonds in these compounds. In the crystal, hydroxyl groups of the 1,3-diazinane ring are linked to water molecules *via* O—H···O hydrogen bonds, leading to a two-molecule aggregate where the water-O accepts these interactions. These are linked into a *zigzag* chain along the *a* axis *via* O—H···O interactions between the water molecules and the phenolic-O atoms. (Fig. 2 and Table 1).

### 2. Experimental

The title compound was prepared as follows: In a 50 ml round bottom flask equipped with a magnetic stir bar, 1 equiv. of 1,3-diamino-2-propanol, 3 equiv. of paraformaldehyde, and 2 equiv. of 2-*tert*-butyl-4-methoxyphenol were combined with 15 ml of methanol. Upon completion of the addition, the reaction mixture was stirred under reflux for 36 h. Then the reflux was stopped, the solvent was removed on a rotary evaporator under vacuum and the residue obtained (Anal. calcd. for C_28_H_42_N_2_O_5_: C 69.14%; H 8.64%; N 5.76%, O 16.46% found C 69.50%; H 8.66%; N 5.77%) was recrystallized from methanol to provide high quality crystals of the title compound (Yield 30.6%. *M*.p. = 393 K).

### 3. Refinement

H atoms bonded to C were positioned geometrically, with C–H = 0.95–0.99 Å and constrained to ride on their parent atoms, with *U*_iso_(H) = 1.5*U*_eq_(C) for methyl H atoms and 1.2*U*_eq_(C) for other H atoms. The non disordered hydroxyl H atoms were refined isotropically, but the disordered ones were refined with *U*_iso_(H) = 1.5*U*_eq_(O). The water H atoms were geometrically positioned with O–H = 0.84 Å and constrained to ride on their parent atom with *U*_iso_(H) = 1.5*U*_eq_(O).

### Crystal data

**Table:**

C_28_H_42_N_2_O_5_·H_2_O	*D*_x_ = 1.215 Mg m^−^^3^
*M**_r_* = 504.65	Mo *K*α radiation, λ = 0.71073 Å
Orthorhombic, *P**n**m**a*	Cell parameters from 25046 reflections
*a* = 8.2629 (5) Å	θ = 1.9–26.5°
*b* = 33.093 (3) Å	µ = 0.09 mm^−^^1^
*c* = 10.0877 (6) Å	*T* = 173 K
*V* = 2758.4 (3) Å^3^	Block, colourless
*Z* = 4	0.25 × 0.23 × 0.16 mm
*F*(000) = 1096	

### Data collection

**Table:**

STOE IPDS II two-circle-diffractometer	2228 reflections with *I* > 2σ(*I*)
ω scans	*R*_int_ = 0.109
Absorption correction: multi-scan (*X-AREA*; Stoe & Cie, 2001)	θ_max_ = 25.0°, θ_min_ = 2.1°
*T*_min_ = 0.975, *T*_max_ = 0.982	*h* = −9→9
22328 measured reflections	*k* = −38→39
2464 independent reflections	*l* = −11→12

### Refinement

**Table:**

Refinement on *F*^2^	0 restraints
Least-squares matrix: full	Hydrogen site location: mixed
*R*[*F*^2^ > 2σ(*F*^2^)] = 0.075	H atoms treated by a mixture of independent and constrained refinement
*wR*(*F*^2^) = 0.140	*w* = 1/[σ^2^(*F*_o_^2^) + (0.0336*P*)^2^ + 2.7349*P*] where *P* = (*F*_o_^2^ + 2*F*_c_^2^)/3
*S* = 1.17	(Δ/σ)_max_ = 0.009
2464 reflections	Δρ_max_ = 0.26 e Å^−^^3^
179 parameters	Δρ_min_ = −0.31 e Å^−^^3^

### Special details

**Table:**

Geometry. All e.s.d.'s (except the e.s.d. in the dihedral angle between two l.s. planes) are estimated using the full covariance matrix. The cell e.s.d.'s are taken into account individually in the estimation of e.s.d.'s in distances, angles and torsion angles; correlations between e.s.d.'s in cell parameters are only used when they are defined by crystal symmetry. An approximate (isotropic) treatment of cell e.s.d.'s is used for estimating e.s.d.'s involving l.s. planes.

### Fractional atomic coordinates and isotropic or equivalent isotropic displacement parameters (Å^2^)

**Table:**

	*x*	*y*	*z*	*U*_iso_*/*U*_eq_	Occ. (<1)
O1	0.4835 (2)	0.17942 (5)	0.52432 (17)	0.0322 (4)	
H1	0.405 (4)	0.1990 (10)	0.546 (3)	0.064 (10)*	
O2	0.1840 (2)	0.03093 (5)	0.5809 (2)	0.0503 (6)	
O3	−0.0101 (4)	0.2500	0.3032 (3)	0.0390 (10)	0.836 (8)
H3	−0.109 (8)	0.2500	0.338 (6)	0.059*	0.836 (8)
O3'	0.253 (2)	0.2500	0.3421 (19)	0.058 (6)*	0.164 (8)
H3'	0.2334	0.2500	0.2606	0.087*	0.164 (8)
N1	0.2068 (2)	0.21393 (5)	0.60143 (18)	0.0261 (4)	
C1	0.1795 (4)	0.2500	0.6823 (3)	0.0264 (7)	
H1A	0.2544	0.2500	0.7589	0.032*	
H1B	0.0672	0.2500	0.7165	0.032*	
C2	0.1058 (4)	0.2500	0.4066 (3)	0.0310 (7)	
H2	0.2162	0.2500	0.3659	0.037*	0.836 (8)
H2'	0.0174	0.2500	0.3389	0.037*	0.164 (8)
C3	0.0894 (3)	0.21229 (6)	0.4924 (2)	0.0302 (5)	
H3A	−0.0217	0.2106	0.5287	0.036*	
H3B	0.1090	0.1879	0.4379	0.036*	
C4	0.2008 (3)	0.17720 (6)	0.6846 (2)	0.0301 (5)	
H4A	0.0874	0.1721	0.7114	0.036*	
H4B	0.2649	0.1818	0.7662	0.036*	
C5	0.2659 (3)	0.14019 (6)	0.6138 (2)	0.0267 (5)	
C6	0.4081 (3)	0.14241 (6)	0.5402 (2)	0.0269 (5)	
C7	0.4776 (3)	0.10750 (6)	0.4830 (2)	0.0272 (5)	
C8	0.3949 (3)	0.07128 (7)	0.5020 (2)	0.0322 (5)	
H8	0.4383	0.0472	0.4648	0.039*	
C9	0.2515 (3)	0.06899 (7)	0.5731 (3)	0.0339 (6)	
C10	0.1861 (3)	0.10328 (7)	0.6299 (2)	0.0301 (5)	
H10	0.0884	0.1018	0.6793	0.036*	
C11	0.6395 (3)	0.10903 (7)	0.4079 (2)	0.0302 (5)	
C12	0.6259 (3)	0.13624 (8)	0.2851 (2)	0.0386 (6)	
H12A	0.7300	0.1368	0.2385	0.058*	
H12B	0.5970	0.1637	0.3126	0.058*	
H12C	0.5422	0.1256	0.2258	0.058*	
C13	0.7719 (3)	0.12506 (8)	0.5006 (2)	0.0375 (6)	
H13A	0.8751	0.1261	0.4530	0.056*	
H13B	0.7822	0.1071	0.5774	0.056*	
H13C	0.7429	0.1523	0.5309	0.056*	
C14	0.6927 (3)	0.06718 (8)	0.3597 (3)	0.0463 (7)	
H14A	0.7957	0.0695	0.3122	0.070*	
H14B	0.6102	0.0561	0.3001	0.070*	
H14C	0.7062	0.0492	0.4360	0.070*	
C15	0.0445 (4)	0.02625 (8)	0.6617 (4)	0.0574 (9)	
H15A	0.0090	−0.0020	0.6594	0.086*	
H15B	−0.0423	0.0437	0.6284	0.086*	
H15C	0.0707	0.0339	0.7531	0.086*	
O1W	−0.3119 (4)	0.2500	0.4322 (3)	0.0694 (9)	
H1W	−0.3666	0.2706	0.4510	0.104*	

### Atomic displacement parameters (Å^2^)

**Table:**

	*U*^11^	*U*^22^	*U*^33^	*U*^12^	*U*^13^	*U*^23^
O1	0.0300 (9)	0.0264 (8)	0.0403 (9)	−0.0030 (7)	0.0049 (8)	0.0005 (7)
O2	0.0512 (12)	0.0248 (9)	0.0750 (14)	−0.0087 (8)	0.0206 (11)	−0.0029 (9)
O3	0.0355 (19)	0.0515 (19)	0.0301 (17)	0.000	−0.0060 (14)	0.000
N1	0.0302 (10)	0.0222 (9)	0.0261 (10)	0.0024 (7)	0.0019 (8)	−0.0003 (7)
C1	0.0272 (17)	0.0238 (15)	0.0281 (17)	0.000	0.0024 (14)	0.000
C2	0.0301 (18)	0.0344 (17)	0.0287 (17)	0.000	−0.0030 (15)	0.000
C3	0.0318 (13)	0.0288 (12)	0.0300 (12)	−0.0011 (9)	−0.0027 (10)	−0.0029 (9)
C4	0.0338 (13)	0.0271 (12)	0.0294 (12)	0.0022 (9)	0.0058 (10)	0.0028 (9)
C5	0.0265 (12)	0.0273 (11)	0.0261 (11)	0.0029 (9)	−0.0001 (9)	0.0029 (9)
C6	0.0253 (11)	0.0270 (11)	0.0283 (12)	−0.0008 (9)	−0.0029 (10)	0.0025 (9)
C7	0.0253 (12)	0.0298 (11)	0.0263 (11)	0.0029 (9)	−0.0035 (10)	0.0002 (9)
C8	0.0352 (13)	0.0258 (11)	0.0356 (13)	0.0035 (9)	0.0011 (11)	−0.0018 (9)
C9	0.0360 (13)	0.0234 (11)	0.0421 (14)	−0.0026 (10)	−0.0004 (12)	0.0026 (10)
C10	0.0265 (12)	0.0300 (12)	0.0337 (13)	0.0000 (9)	0.0027 (10)	0.0044 (9)
C11	0.0269 (12)	0.0349 (12)	0.0290 (12)	0.0031 (10)	0.0010 (10)	−0.0020 (10)
C12	0.0331 (14)	0.0535 (15)	0.0292 (13)	0.0014 (11)	0.0031 (11)	0.0023 (11)
C13	0.0243 (12)	0.0557 (15)	0.0324 (13)	0.0027 (11)	−0.0006 (10)	−0.0006 (11)
C14	0.0408 (16)	0.0419 (14)	0.0563 (17)	0.0058 (12)	0.0137 (14)	−0.0075 (13)
C15	0.0542 (18)	0.0335 (14)	0.085 (2)	−0.0102 (13)	0.0229 (17)	0.0044 (14)
O1W	0.0420 (18)	0.096 (3)	0.070 (2)	0.000	0.0107 (17)	0.000

### Geometric parameters (Å, º)

**Table:**

O1—C6	1.383 (3)	C5—C10	1.397 (3)
O1—H1	0.94 (3)	C6—C7	1.413 (3)
O2—C9	1.380 (3)	C7—C8	1.393 (3)
O2—C15	1.420 (3)	C7—C11	1.538 (3)
O3—C2	1.417 (4)	C8—C9	1.388 (3)
O3—H3	0.89 (6)	C8—H8	0.9500
O3'—C2	1.378 (19)	C9—C10	1.381 (3)
O3'—H3'	0.8375	C10—H10	0.9500
N1—C1	1.463 (2)	C11—C14	1.532 (3)
N1—C3	1.468 (3)	C11—C13	1.534 (3)
N1—C4	1.478 (3)	C11—C12	1.536 (3)
C1—N1^i^	1.463 (2)	C12—H12A	0.9800
C1—H1A	0.9900	C12—H12B	0.9800
C1—H1B	0.9900	C12—H12C	0.9800
C2—C3^i^	1.525 (3)	C13—H13A	0.9800
C2—C3	1.525 (3)	C13—H13B	0.9800
C2—H2	1.0000	C13—H13C	0.9800
C2—H2'	1.0000	C14—H14A	0.9800
C3—H3A	0.9900	C14—H14B	0.9800
C3—H3B	0.9900	C14—H14C	0.9800
C4—C5	1.516 (3)	C15—H15A	0.9800
C4—H4A	0.9900	C15—H15B	0.9800
C4—H4B	0.9900	C15—H15C	0.9800
C5—C6	1.392 (3)	O1W—H1W	0.8401
			
C6—O1—H1	106 (2)	C8—C7—C6	116.6 (2)
C9—O2—C15	117.4 (2)	C8—C7—C11	121.5 (2)
C2—O3—H3	109 (4)	C6—C7—C11	121.83 (19)
C2—O3'—H3'	107.1	C9—C8—C7	122.5 (2)
C1—N1—C3	110.24 (19)	C9—C8—H8	118.8
C1—N1—C4	110.44 (18)	C7—C8—H8	118.8
C3—N1—C4	111.89 (17)	O2—C9—C10	124.6 (2)
N1—C1—N1^i^	109.3 (2)	O2—C9—C8	115.1 (2)
N1—C1—H1A	109.8	C10—C9—C8	120.2 (2)
N1^i^—C1—H1A	109.8	C9—C10—C5	119.0 (2)
N1—C1—H1B	109.8	C9—C10—H10	120.5
N1^i^—C1—H1B	109.8	C5—C10—H10	120.5
H1A—C1—H1B	108.3	C14—C11—C13	107.6 (2)
O3'—C2—C3^i^	110.3 (4)	C14—C11—C12	107.2 (2)
O3—C2—C3^i^	110.95 (19)	C13—C11—C12	110.0 (2)
O3'—C2—C3	110.3 (4)	C14—C11—C7	112.09 (19)
O3—C2—C3	110.95 (19)	C13—C11—C7	109.33 (19)
C3^i^—C2—C3	109.9 (3)	C12—C11—C7	110.68 (19)
O3—C2—H2	108.3	C11—C12—H12A	109.5
C3^i^—C2—H2	108.3	C11—C12—H12B	109.5
C3—C2—H2	108.3	H12A—C12—H12B	109.5
O3'—C2—H2'	108.8	C11—C12—H12C	109.5
C3^i^—C2—H2'	108.8	H12A—C12—H12C	109.5
C3—C2—H2'	108.8	H12B—C12—H12C	109.5
N1—C3—C2	109.6 (2)	C11—C13—H13A	109.5
N1—C3—H3A	109.7	C11—C13—H13B	109.5
C2—C3—H3A	109.7	H13A—C13—H13B	109.5
N1—C3—H3B	109.7	C11—C13—H13C	109.5
C2—C3—H3B	109.7	H13A—C13—H13C	109.5
H3A—C3—H3B	108.2	H13B—C13—H13C	109.5
N1—C4—C5	112.64 (18)	C11—C14—H14A	109.5
N1—C4—H4A	109.1	C11—C14—H14B	109.5
C5—C4—H4A	109.1	H14A—C14—H14B	109.5
N1—C4—H4B	109.1	C11—C14—H14C	109.5
C5—C4—H4B	109.1	H14A—C14—H14C	109.5
H4A—C4—H4B	107.8	H14B—C14—H14C	109.5
C6—C5—C10	120.4 (2)	O2—C15—H15A	109.5
C6—C5—C4	120.53 (19)	O2—C15—H15B	109.5
C10—C5—C4	118.9 (2)	H15A—C15—H15B	109.5
O1—C6—C5	119.23 (19)	O2—C15—H15C	109.5
O1—C6—C7	119.6 (2)	H15A—C15—H15C	109.5
C5—C6—C7	121.2 (2)	H15B—C15—H15C	109.5
			
C3—N1—C1—N1^i^	−63.1 (3)	C5—C6—C7—C11	−176.6 (2)
C4—N1—C1—N1^i^	172.70 (17)	C6—C7—C8—C9	0.0 (3)
C1—N1—C3—C2	59.1 (3)	C11—C7—C8—C9	177.8 (2)
C4—N1—C3—C2	−177.6 (2)	C15—O2—C9—C10	−5.2 (4)
O3'—C2—C3—N1	67.0 (8)	C15—O2—C9—C8	174.9 (2)
O3—C2—C3—N1	−177.8 (2)	C7—C8—C9—O2	179.1 (2)
C3^i^—C2—C3—N1	−54.8 (3)	C7—C8—C9—C10	−0.9 (4)
C1—N1—C4—C5	−166.7 (2)	O2—C9—C10—C5	−179.5 (2)
C3—N1—C4—C5	70.1 (2)	C8—C9—C10—C5	0.5 (4)
N1—C4—C5—C6	44.2 (3)	C6—C5—C10—C9	0.7 (3)
N1—C4—C5—C10	−139.5 (2)	C4—C5—C10—C9	−175.5 (2)
C10—C5—C6—O1	179.2 (2)	C8—C7—C11—C14	0.1 (3)
C4—C5—C6—O1	−4.6 (3)	C6—C7—C11—C14	177.9 (2)
C10—C5—C6—C7	−1.7 (3)	C8—C7—C11—C13	−119.1 (2)
C4—C5—C6—C7	174.6 (2)	C6—C7—C11—C13	58.7 (3)
O1—C6—C7—C8	−179.5 (2)	C8—C7—C11—C12	119.7 (2)
C5—C6—C7—C8	1.3 (3)	C6—C7—C11—C12	−62.6 (3)
O1—C6—C7—C11	2.6 (3)		

Symmetry code: (i) *x*, −*y*+1/2, *z*.

### Hydrogen-bond geometry (Å, º)

**Table:**

*D*—H···*A*	*D*—H	H···*A*	*D*···*A*	*D*—H···*A*
O1—H1···N1	0.94 (3)	1.80 (3)	2.671 (2)	153 (3)
O3—H3···O1*W*	0.89 (6)	1.93 (6)	2.813 (4)	174 (5)
O1*W*—H1*W*···O1^ii^	0.84	2.19	3.029 (2)	173

Symmetry code: (ii) *x*−1, −*y*+1/2, *z*.
